# Post-Transcriptional Modification Integration for Ligand–Receptor Cellular Network Inference

**DOI:** 10.1016/j.mcpro.2025.101493

**Published:** 2025-12-19

**Authors:** Pierre Giroux, Morgan Maillard, Jacques Colinge

**Affiliations:** 1 IRCM, Institut de Recherche en Cancérologie de Montpellier, INSERM U1194, Montpellier, France; 2 Université de Montpellier, Montpellier, France; 3 ICM, Institut régional du Cancer de Montpellier, Montpellier, France

**Keywords:** ligand–receptor interactions, post-translational modifications, proteomics data integration

## Abstract

Cell–cell communications are widely explored to understand tissue homeostasis and diseases. Numerous computational tools have been developed to infer cellular interactions from transcriptomic or proteomic expression data. However, proteins often carry post-translational modifications (PTMs) that can induce conformational switches and alter their functional properties. A key challenge remains to incorporate PTM data in the inference and analysis of cellular interactions. Here, we propose an extension of our previously published tool BulkSignalR to integrate PTM information in ligand–receptor interactions and downstream pathway predictions. This new functionality is compatible with bulk and single-cell data, and it supports all types of PTMs. Based on two illustrative datasets, we show that this new feature provides deeper insights into biological pathway regulation and that PTM integration helps reduce false-positive results occasionally produced by standard approaches.

## Highlights

• Extending BulkSignalR to integrate post-translational modifications (PTMs) into ligand–receptor interaction inference.
• PTM integration improves accuracy and confidence in pathway activation prediction.
• Application to two cancer datasets reveals context-specific signaling.
• Framework supports all types of PTMs.

## Graphical Abstract

**Figure:**
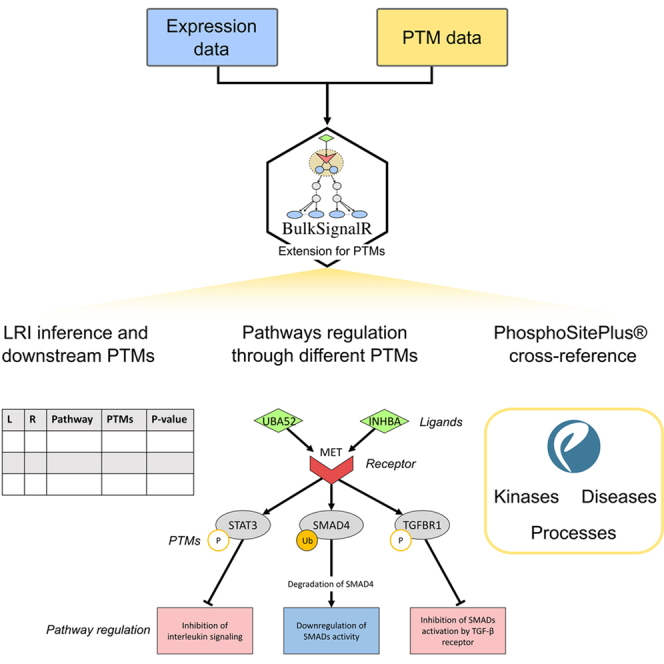

## In Brief

We developed an extension of BulkSignalR to integrate post-translational modification data in the inference of ligand–receptor interactions and downstream signaling pathways. Applied to laryngeal squamous cell carcinoma and renal carcinoma datasets, this approach refines pathway activation analysis, reduces false positives, and reveals context-dependent signaling mechanisms. By combining proteomic and post-translational modification data, it provides a more mechanistic understanding of cellular communication and regulatory processes across biological contexts.

Cell–cell communication through ligand–receptor interactions (LRIs) plays a major role in homeostasis, tissue development, and diseases. Cellular networks mediated by LRIs have been widely studied recently, and several tools were developed to predict such networks from transcriptomics or proteomics data by combining either molecule coexpression or correlation analyses with curated interaction databases, such as Kyoto Encyclopedia of Genes and Genomes or Reactome (1, 2, 3, 4). While transcriptomics is commonly used, notably at single-cell resolution, proteomics usage remained more marginal. This is understandable, especially at single-cell resolution, where proteomics is just emerging (5) but quite regrettable since proteomics offers a more accurate view of biological samples (5, 6, 7, 8) regarding pathway regulation. This is especially true considering the fundamental layer of regulation provided by post-translational modifications (PTMs), which is inaccessible to transcriptomic technologies (9). Indeed, 30% to 65% of proteins may be phosphorylated in humans, and phosphorylation of protein residues plays critical roles in the regulation of many biological pathways, like cell proliferation, growth, and apoptosis (10, 11). Glycosylation of protein affects protein folding, distribution, and activity, whereas ubiquitination usually leads to protein inactivation and degradation (12, 13). PTM data must then be able to be integrated into cellular network analysis to have a realistic overview of pathway activity. Currently, to the best of our knowledge, no LRI inference tool takes PTMs into account, with the exception of one case where single-cell RNA-sequencing data are necessary, and LRIs are only annotated by phosphoproteomics data (14).

We introduce a new method, based on our previous BulkSignalR package (15) that is able to infer LRIs from bulk expression data. This extension can now combine bulk expression proteomics and PTM-specific proteomics data to infer cellular pathways. It is applicable to all possible PTMs (phosphorylation, ubiquitination, acetylation, etc.).

We applied this computational framework to study cellular networks in cancer. Tumor progression and therapeutic resistance are closely linked to complex interactions within the tumor microenvironment (TME), which includes not only cancer cells but also immune cell populations, cancer-associated fibroblasts, and neo blood vessels. The TME has been reported to be involved in cancer progression, invasion, and resistance, and the signaling pathways associated with these cellular processes are mediated by various PTMs (16). Understanding cellular interactions and PTM changes occurring in the TME is crucial to decipher cancer biology and combat tumors, as exemplified by immunotherapies targeting TME immune checkpoints or antiangiogenic therapies targeting neo vessel development (17, 18, 19).

Here, we describe and discuss the application of the PTM-extended BulkSignalR tool using two public cancer datasets as an illustration, with a focus on phosphorylation and ubiquitination modifications. The method is generalizable to any other proteomics dataset containing potentially interacting cell populations. The integration of PTMs in LRI analysis will offer crucial insight into all areas of biology.

## Experimental Procedures

### Experimental Design and Statistical Rationale

Lung squamous cell carcinoma (LSCC) data were downloaded from Clinical Proteomic Tumor Analysis Consortium (CPTAC) Pan-Cancer Data portal (20). We used LSCC proteome data processed by the University of Michigan team's pipeline and then postprocessed by the Mount Sinai team's pipeline, LSCC phosphoproteome data processed by the University of Michigan team's pipeline and then postprocessed by the Mount Sinai team's pipeline and LSCC ubiquitylome data processed by Common Data Analysis pipelines. In that study, 108 treatment-naive primary LSCC tumors and 99 matched adjacent normal tissues were collected and processed for deep proteomic and PTM profiling using LC–MS/MS after peptide digestion, fractionation, and enrichment steps (*e.g.*, phosphopeptide enrichment). Quantification was achieved *via* a tandem mass tag–based approach with stringent quality filtering of proteins and PTMs detected across samples. CPTAC Pan-Cancer clinical and metadata were downloaded from the same portal. Phosphorylation level in a given sample was normalized by the corresponding protein expression in the same sample (phosphoprotein or phosphosite level divided by protein expression level). Ubiquitination levels were normalized using the same method.

Clear cell renal cell carcinoma (ccRCC) proteomic and phosphoproteomic data were downloaded from the ProteomeXchange database ProteomeCentral, ID PXD042844. In that study, fresh-frozen tissue samples were processed by protein extraction and tryptic digestion, peptides were fractionated and enriched for phosphopeptides where appropriate, and analyzed by high-resolution LC–MS/MS. Quantification was performed in a label-free manner, retaining proteins or phosphosites detected in at least 25% of samples. The dataset comprises 115 tumor samples with matched adjacent tissue for global proteomics and 66 samples for phosphoproteomics. Clinical and metadata were obtained from supplemental data provided by Zhang *et al*. (21). Phosphorylation levels were normalized with the method described above.

The files “Regulatory_sites.gz,” “Kinase_Substrate_Dataset.gz,” and “Disease-associated_sites.gz” were downloaded from PhosphoSitePlus (https://www.phosphosite.org/staticDownloads; accessed September 30, 2025).

### Implementation and Visualization

In the same way as the original BulkSignalR software, this extension is implemented in R following an S4 object-oriented approach. The software is available with documentation from GitHub (https://github.com/girouxpierre/PTMSignalR) and Zenodo (doi.org/10.5281/zenodo.15882213). Interaction networks were displayed by the tool and exported to be modified on the Cytoscape software (cytoscape.org). Comparison of pathway significance obtained with expression and PTM data can be performed from BulkSignalR outputs and visualized using an R script available on GitHub.

## Results

### Extending BulkSignalR Databases to PTMs

**Fig. 1:**
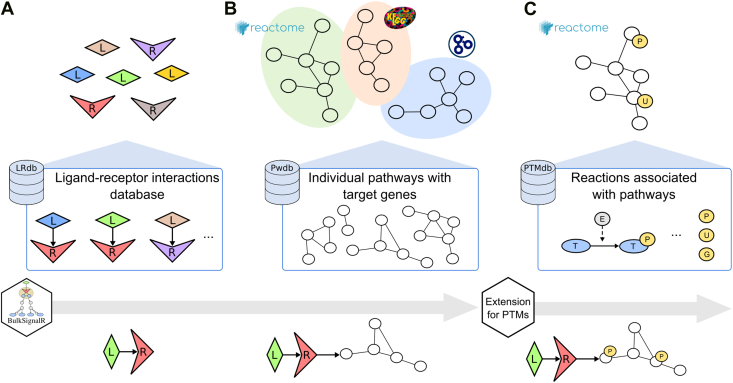
**Databases created for BulkSignalR and its extension for PTMs.***A*, known ligand (L) and receptor (R) interactions were aggregated in LR*db*. *B*, biological pathways and associated target genes (T) were extracted from Reactome and Gene Ontology biological process databases. *C*, phosphorylation (P), ubiquitination (U), and glycosylation (G) events occurring in pathways according to Reactome were integrated in a specific database containing the modified residues. PTM, post-translational modification.

To perform its computations, BulkSignalR relies on several reference databases, which are described in detail in the original publication (22). BulkSignalR is compatible with transcriptomic and proteomic expression data. In the context of this report, we will focus on protein-level analyses. Briefly, a global, partially directed network of intracellular molecular interactions is assembled, combining Reactome (23) and Kyoto Encyclopedia of Genes and Genomes (24) database interactions. This intracellular network is then connected to LR*db*, our precompiled database of known interactions (25). Potential LRIs are taken from the LR*db* database, and each receptor is linked to downstream protein targets based on cellular pathways defined as lists of proteins according to Reactome (23) and Gene Ontology biological processes (26). Targets of a given receptor within a given pathway are defined as proteins accessible by following the intracellular network corresponding to the chosen pathway. These target proteins are annotated as regulated by the pathway activity (Fig. 1, *A* and *B*).

To include data related to PTMs, we needed an additional reference database of protein modification events connected with biological pathways (Fig. 1*C*). This resource was assembled from the 2024 release of the Reactome Neo4j graph database, which provided structured biochemical reaction data. We queried the database using Cypher (Reactome’s native graph query language) to extract all the reactions in which proteins with annotated PTMs were present either as input or output entities. For each reaction, we retrieved the positions of the modified residue, the type of PTM (phosphorylation, ubiquitination, or glycosylation), and the catalyzing enzyme. Protein complexes are also defined, but while proteins in a complex are all connected to each other, some gigantic complexes may potentially cause excessive connectivity. To address this problem, we set a threshold on complex sizes (currently at 10), beyond which the complexes are ignored. Reactions annotated as candidate interactions were also excluded to minimize potential false positives. A custom Python script was developed to identify differential PTM positions on individual residues across reactions. Namely, for a given protein and residue, if a change in PTM position was observed between the reactant and product states, we supposed a functional link between the catalyzing enzymes and the target modification site. This approach enabled us to distinguish modification-specific directions, such as phosphorylation *versus* dephosphorylation, and to assign exact amino acid positions for each PTM event. This pipeline resulted in three curated datasets of PTM-specific enzymatic reactions associated with known Reactome pathways. These datasets contain 6805, 707, and 324 identified reaction-pathway pairs for phosphorylation, ubiquitination, and glycosylation, respectively (Supplemental Table S1).

### Extending BulkSignalR Algorithms to PTMs

**Fig. 2:**
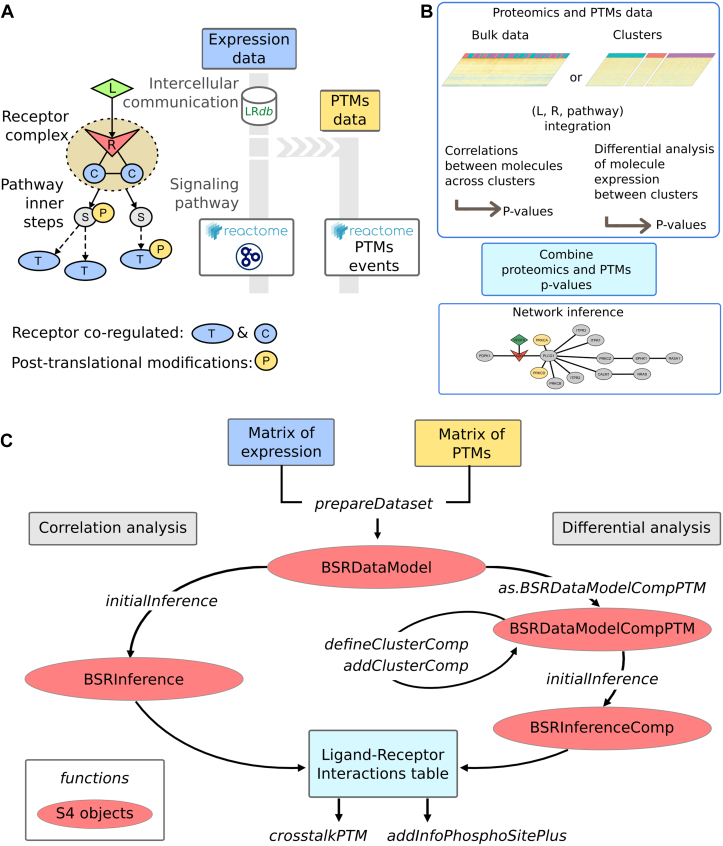
**Integration of PTM data in BulkSignalR.***A*, model of LRI with a pathway involving PTMs downstream of the receptor. C, proteins forming a complex with the receptor protein; S, intermediary protein; L, ligand; P, PTM; R, receptor; and T, targets, that is, proteins whose expression or modification is regulated by the pathway. Known LRIs are taken from LRdb or supplied by the user. Pathways and their regulated proteins are defined by Reactome and Gene Ontology biological process (GOBP). PTMs occurring in pathways are defined by Reactome or provided by the user. *B*, parallel representation of expression and PTM information, LRI inference by correlation or cluster differential analysis. The results from both data types are combined in a unique table. Observed PTM events are represented in *orange* for residue addition (actual increase) and *pale yellow* for subtraction (actual decrease). Border colors represent the expected events according to the reference database. Contradictions will be highlighted by borders different from the inner color. *C*, schematic flowchart of the BulkSignalR extension. Input data (expression and PTM matrices) can be processed either with correlation or differential mode after dataset preparation. Both approaches produce an LRI inference table, which can be explored with additional functions specific to PTM analysis. LRI, ligand–receptor interaction; PTM, post-translational modification.

The standard version of BulkSignalR infers LRIs from bulk proteomic data represented in a table, where proteins are in rows and samples in columns. By default, BulkSignalR relies on correlation analysis to find ligand–receptor pairs along with regulated target proteins downstream of the receptor in a pathway (Fig. 2*A*). To add quantitative PTM information, we stored PTM data in a second, parallel table (Fig. 2*B*). PTM information for proteins not detected at the expression level was discarded since our aim was to integrate PTMs as complementary information to downstream pathways predicted based on expression data. Beyond its correlation-based default analysis mode, BulkSignalR can work with clusters of samples and compare them 2-by-2 to find cellular communications that are differentially modulated. In that case, differential expression and associated *p* values for the involved molecules are used instead of *p* values obtained from correlation analyses. This second mode has also been implemented in the PTM extension (Fig. 2*B*). In practice, we found the latter mode more effective with PTMs. Correlation analysis may help to identify LRIs that would not be regulated with a shift in protein expression, but we found the results more complex to interpret for PTMs. Accordingly, and for the sake of simplicity, we only describe the differential expression mode in the sequel. Details about correlation analysis are available from the BulkSignalR article (22).

In terms of implementation, the original BulkSignalR was developed in R following an S4 object-oriented approach. To integrate PTM data, we preserved the same scaffold by implementing daughter classes to store and process the PTM-specific data. This enabled us to maintain access to the rich set of functionalities for LRI additional analyses and graphic functions provided by BulkSignalR (22). The newly introduced daughter classes added the representation of PTM quantitative data. The BulkSignalR pipeline is detailed in Figure 2*C*.

The general strategy behind the BulkSignalR statistical model to assess LRIs combines statistical evidence for coexpression, or coregulation, of the ligand and the receptor, along with a number of receptor targets downstream of a pathway containing the receptor. In a table containing protein expression across multiple samples, we want to compare two groups or clusters of samples, *A* and *B*. By applying a statistical test, each detected protein is associated with a fold change and a *p* value in this comparison. By default, we performed a simple two-sided Wilcoxon test, but the users can use any test of their own choice to assess differential expression, such as limma (27) or significance analysis of microarrays (28) which are commonly used in proteomics. Significance of the regulation of the ligand, the receptor, and its targets across the two clusters is reported by three distinct *p* values. To capture the notion that not all the targets in a pathway might be detected or actually regulated, pathway significance relies on the computation of a rank statistic of the sorted target *p* values (22), and the pathway *p* value is provided by the rank statistic *p* value. Last, an overall *p* value is computed for each LRI by simply multiplying the ligand, receptor, and pathway *p* values, assuming independence for simplicity. Multiple hypothesis correction is applied with Benjamini–Hochberg as the default method. The ligand is imposed to display increased expression in the second cluster. By default, the receptor is also imposed positive regulation, though negative, inhibitory regulation is an available option. The target regulation sign is not imposed by default, but positive regulation can be imposed.

**Fig. 3:**
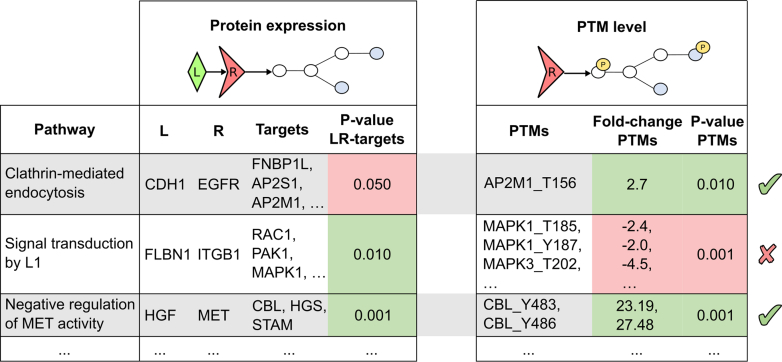
**Typical output from BulkSignalR PTM extension.***Left*, original, expression-based output with ligands, their receptors, the receptor downstream pathways, and the targets in the latter pathway. *Right*, PTM-based output. Pathways associated with ambiguous *p* values from expression data, for example, the clathrin-mediated endocytosis pathway, can be assessed by checking for significant and coherent PTM regulation and can be selected with greater confidence than initially. Conversely, pathways with significant expression-associated *p* values can be discarded by incoherent PTM regulation, for example, signal transduction by the L1 pathway where PTMs should be upregulated. Significant *p* values and coherence in PTM regulation lead to increased confidence in pathway activation, for example, negative regulation of MET activity. MET, MET proto-oncogene, receptor tyrosine kinase; PTM, post-translational modification.

The PTM-extended BulkSignalR algorithm first infers LRIs by employing expression proteomics data only, as in the original tool, but imposing no threshold on significance. Then, each LRI, including its downstream pathway targets, is refined by searching for PTMs that are coherent with our PTM reference database. This strategy was motivated by the usual sparsity of PTM data, which prevents any reliable prediction of LRIs based solely on these data. The PTM refinement step checks whether enzymes annotated in the pathway were detected (in expression data) along with their substrate, and the corresponding substrate PTM was regulated. PTM regulation concordance with the reference database, for example, increased phosphorylation after a kinase, or decreased after a phosphatase, is recorded, but incoherencies are not discarded by default to consider errors in the reference or more complex regulatory processes than a single enzyme. PTM variations are also associated with statistical parameters (a difference delta value and *p* value) comparing the two clusters of samples, which allows us to compute a pathway PTM regulation *p* value by rank statistics, as with expression data. Finally, a threshold is applied to the LRI *p* value based on expression only and on the PTM *p* value (Fig. 3). The conditions on the two *p* values can be set such that an LRI that is not significant based on expression data only can be predicted with greater confidence by significant PTM regulation. Alternatively, the user can also just further multiply PTM and non-PTM *p* values to obtain a similar selection.

Unannotated PTMs can also be analyzed with the aim of investigating their changes within a specific pathway. For this purpose, the user can substitute the provided PTM database with a list of PTMs and corresponding pathways of interest. The PTM changes will appear in these signaling pathways with their corresponding *p* value.

Two additional functions were implemented specifically for PTM analyses. The first one allows to explore crosstalk between different types of PTMs. It integrates the results of two analyses performed with different types of PTMs (*e.g.*, phosphorylation and ubiquitination) and allows to identify (i) inferred signaling pathways involving modifications of the two types of PTMs, (ii) inferred LRIs involving downstream modifications of the two types of PTMs, and (iii) proteins modified by the two types of PTMs. The second function extracts, for the PTMs identified with the BulkSignalR analysis, additional information from the PhosphoSitePlus database (29). PhosphoSitePlus is one of the most complete resources about PTMs and includes data from numerous research articles. When it is available, the extracted information includes for each PTM: the corresponding Site_Group_ID in PhosphoSitePlus, the corresponding kinase, the modified domain, biological functions of the modified protein, biological processes, associated diseases, alteration of the PTM in these diseases (increased/decreased), and additional notes about the implication of the PTM in diseases. These methods enable a deeper understanding and more accurate analysis of the identified pathways and modifications.

To provide additional flexibility, while PTMs are used at their exact position by default, it is also possible to switch to a global protein mode. In that case, when several PTMs are present on a single protein, the most extreme regulation is used to replace all the individual *p* values.

### Application to Lung Squamous Cell Carcinoma

To demonstrate the applicability of our novel method, we analyzed expression proteomics and PTM data from the National Cancer Institute’s CPTAC. The dataset included the proteomes, phosphoproteomes, and ubiquitylomes of different cancer types. We analyzed the LSCC data that included 99 patient tumor samples. The dataset contained 10,909 proteins, 5145 phosphoproteins, 21,319 phosphosites, 2164 ubiquinated proteins, and 6966 ubiquination sites. Our phosphoproteomic analysis enabled to identify 425 cellular pathways containing active phosphosites and associated kinase proteins. Similarly, we found 156 pathways with ubiquitination data. To distinguish changes in PTM levels from variations in basal protein abundance, phosphoproteomics and ubiquitination data were normalized by protein expression. Indeed, because CPTAC uses tandem mass tag labeling, phosphopeptides and unmodified peptides can differ in ionization efficiency and reporter-ion response. In addition, the phosphopeptide fractions are enriched from a larger total protein input than the aliquots used for the global proteome run. Thus, the “phosphosite/protein” ratio we compute does not represent an absolute stoichiometry but a relative measure of phosphorylation that is scaled to the abundance of the parent protein. This normalization, similar to that performed in the original article for acetylation and ubiquitination data, reduces bias because of differences in protein expression between samples and allows comparison of phosphorylation levels on a per-protein basis.

CPTAC LSCC samples were annotated with xCell scores (30) that estimated the abundance of different cell populations or cellular compositions, that is, cancer-associated fibroblasts (CAFs), immune, microenvironment, and stroma. xCell defines the stroma by the combination of adipocytes, endothelial cells, and fibroblasts, and the microenvironment score by the sum of immune and stromal scores. We analyzed LRIs with respect to the four scores (Supplemental Tables S2 and S3). We only report here the results based on the CAFs and immune scores. In both cases, we defined two extreme groups of samples with respect to the xCell score: top 20%, for example, CAF-high, and bottom 20%, for example, CAF-low.

**Fig. 4:**
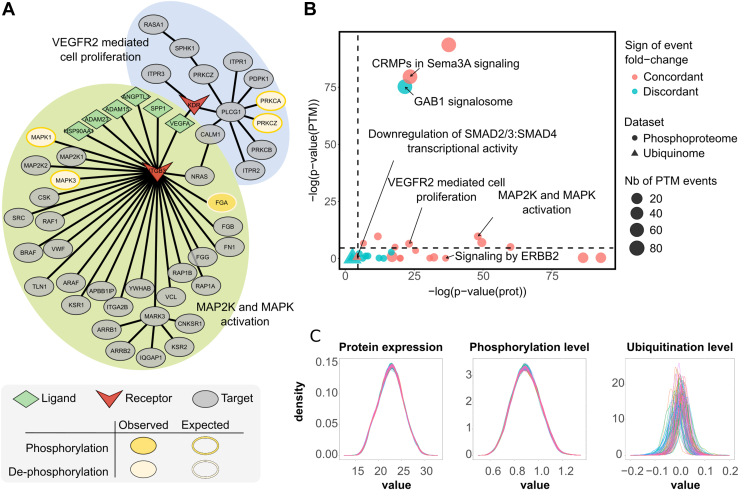
**Expression and PTM data analysis.***A*, pathway significance obtained with expression and PTM data in CAFs low *versus* high clusters. *Dashed lines* represent 0.05 *p* value thresholds. Each dot represents a pathway involving phosphorylation (*circle*) or ubiquitination (*triangle*) events. The size of the dots is proportional to the number of PTMs occurring in the pathway. In *blue*, pathways with PTM addition or subtraction concordant with Reactome annotations. In *red*, the nonconcordant cases. All the triples (ligand, receptor, and pathway) sharing the same pathway were merged and averaged in a unique dot. *B*, selected pathways as obtained with BulkSignalR extension using proteomic and phosphoproteomic data in low-CAF *versus* high-CAF clusters. MAPK1, MAPK3, PRKCA, and PRKCD were observed to be less phosphorylated in the low-CAF cluster than in the high-CAF cluster, although the VEGFR-mediated cell proliferation and MAP2K and MAPK activation pathways should lead to their phosphorylation. On the contrary, fibrinogen alpha chain (FGA) was found more phosphorylated in the low-CAF cluster, though dephosphorylation was expected. *C*, protein expression, phosphorylation, and ubiquitination level distributions across all 99 patients. Due to the differences in enrichment and normalization methods, intensity distributions are on different scales. Ubiquitin-remnant enrichment produces many low-abundance peptides and fewer identifications per sample, which explains the higher apparent noise relative to the global proteome and phosphoproteome datasets. CAF, cancer-associated fibroblast; MAPK, mitogen-activated protein kinase; MAP2K, MAP kinase kinase; PTM, post-translational modification; VEGFR, vascular endothelial growth factor receptor.

The CAF-low (CAF xCell score <4.10^−18^) *versus* CAF-high (CAF xCell score >0.12) comparison revealed high-confidence predictions for the MAP kinase kinase (MAP2K) and mitogen-activated protein kinase (MAPK) activation, and vascular endothelial growth factor receptor 2 (VEGFR2)-mediated cell proliferation pathways in CAF-low samples (*p* values 10^−26^ and 10^−13^, respectively), both pathways associated with VEGFA ligand. However, as these pathways are associated with CAF-driven cancer invasion and proliferation (31, 32, 33), their predicted activation in tissues with low CAF abundance may be biologically questionable, given that no other significant differences were detected in mutation proportions and only slight differences were observed in the xCell scores of other cell types (Supplemental Table). Integrating phosphorylation information in these pathways revealed lower phosphorylation of MAPK1 and MAPK3 proteins in CAF-low samples, negative delta values, and strong statistical significance (*p* = 10^−5^) for the two amino acids (Y-187 and Y-204) expected to be phosphorylated in MAP2K and MAPK activation pathway (Fig. 4*A*). At the protein expression level, the same dynamics were observed for PRKCA and PRKCZ protein phosphorylation in the VEGFR2-mediated cell proliferation pathway (*p* = 0.09). Therefore, although proteins involved in these pathways appear overexpressed in CAF-low samples based on expression data, they show reduced phosphorylation in our PTM analysis. This suggested that the pathways were not activated, which was more consistent with the low abundance of CAFs in the tissue. Twenty additional pathways were found to be associated with phosphorylation events. Some showed concordance with expected phosphorylation patterns, strengthening confidence in their actual activation, whereas others were discordant (Fig. 4*B*). Among the concordant cases, the combined expression-PTM analyses enabled increasing confidence in low-regulated pathways. Of note, differences in *p* values must be related to differences of dynamic range and quality between proteomics and phosphoproteomics data.

**Fig. 5:**
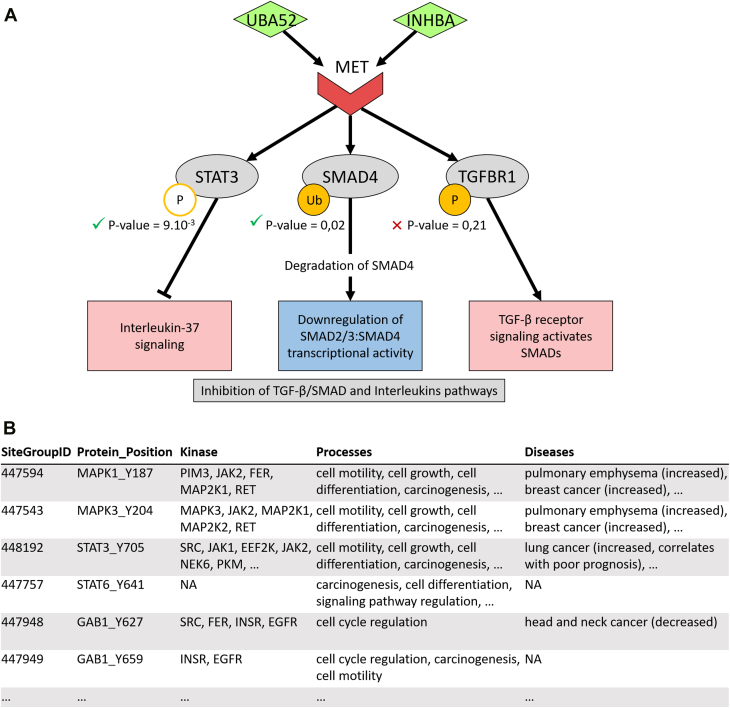
**Results obtained with new functions dedicated to PTM analysis in immune-low *versus* immune-high clusters.***A*, selected proteins and pathways regulated by MET receptor interactions through different types of PTMs. *Orange circles*: PTM events, P stands for phosphorylation, Ub for ubiquitination. *White circles* with *orange outline*: dephosphorylation or less abundant phosphorylation events. *Pale red*: inhibited pathways. *Pale blue*: activated pathways. Annotated *p* values correspond to the ones obtained from the PTM-integrating analysis. All expression-based *p* values were significant (<10^-13^). *B*, selected phosphosite annotations from the PhosphoSitePlus database. MET, MET proto-oncogene, receptor tyrosine kinase; PTM, post-translational modification.

The same procedure was applied to compare immune-low (Immune xCell score <0.06) and immune-high (Immune xCell score >0.28) samples. In interleukin signaling pathways, signal transducer and activator of transcription 3 (STAT3) and STAT6 showed reduced phosphorylation in the immune-low cluster, with a negative delta value and reasonable significance (*p* = 0.01) for the two amino acids (Y-705 and Y-641) expected to be phosphorylated. This suggested pathway inactivation. Moreover, although ubiquitination data appear more heterogeneous between samples and lower signals (Fig. 4*C*), ubiquitination information could still be integrated in the analysis of the downregulation of mother against decapentaplegic (SMAD)2/3–SMAD4 transcriptional activity pathway. In the low-immune cluster, the SMAD4-K385 amino acid had a positive delta value and good significance given the data quality (*p* = 0.02), suggesting SMAD4 protein ubiquitination and subsequent degradation. This event could be translated into SMAD2/3–SMAD4 pathway inactivation in these samples. Therefore, we could predict with higher confidence the downregulation of SMAD2/3–SMAD4 transcriptional activity pathway, a process known to be associated with an immunosuppressive microenvironment (34, 35). The extraction of LRIs involving the two different PTMs in their downstream pathways was performed with a dedicated function of the BulkSignalR extension. It revealed the implication of two different LRIs involving the same receptor (UBA52/MET proto-oncogene, receptor tyrosine kinase [MET] and INHBA/MET) in the regulation of interleukin and SMAD pathways in the immune-low cluster. It appeared that these interactions are also associated with the SMAD activation by transforming growth factor beta (TGF-β) receptor signaling, a pathway predicted using expression data. Nonetheless, the expected phosphorylation of TGFBR1-Y165 in this pathway is not statistically significant (*p* = 0.21) and, thus, allows us to confirm the inhibition of the SMAD pathway downstream of the MET receptor in the immune-low group (Fig. 5*A*).

These results are concordant with the findings of the original publication, which highlights the presence of STAT3-mediated interleukin signaling pathways in immune-high samples. The original analysis explored the association between the regulation of SMAD-related pathways and different immune clusters but with no significant result. Our approach allowed us to statute on this association. Finally, we explored the functional relevance of identified PTMs by crossreferencing them with the PhosphoSitePlus database. Numerous phosphorylation sites analyzed in the predicted signaling pathways were found to be associated with cancer-related processes or are known to be dysregulated in different cancer types (Fig. 5*B*). This additional information may contribute to a more detailed investigation of the biological mechanisms involved and support the relevance of this type of LRI analysis in this context.

### Exploring Cellular Interactions in Renal Cancer Cells

To evaluate the biological interpretability of LRI predictions in a clinical setting, we applied our method to proteomic and phosphoproteomic data from 66 patients with ccRCC treated with sunitinib, a promiscuous tyrosine kinase inhibitor. This dataset, published by Zhang *et al*. (21), included pretreatment tumor samples that were clustered according to the TME composition and response profiles. Due to its limited coverage (5998 proteins and 6199 phosphosites), only 44 Reactome pathways could be mapped using our filtering criteria. Phosphoprotein levels were normalized to protein abundance before BulkSignalR analysis as aforementioned.

**Fig. 6:**
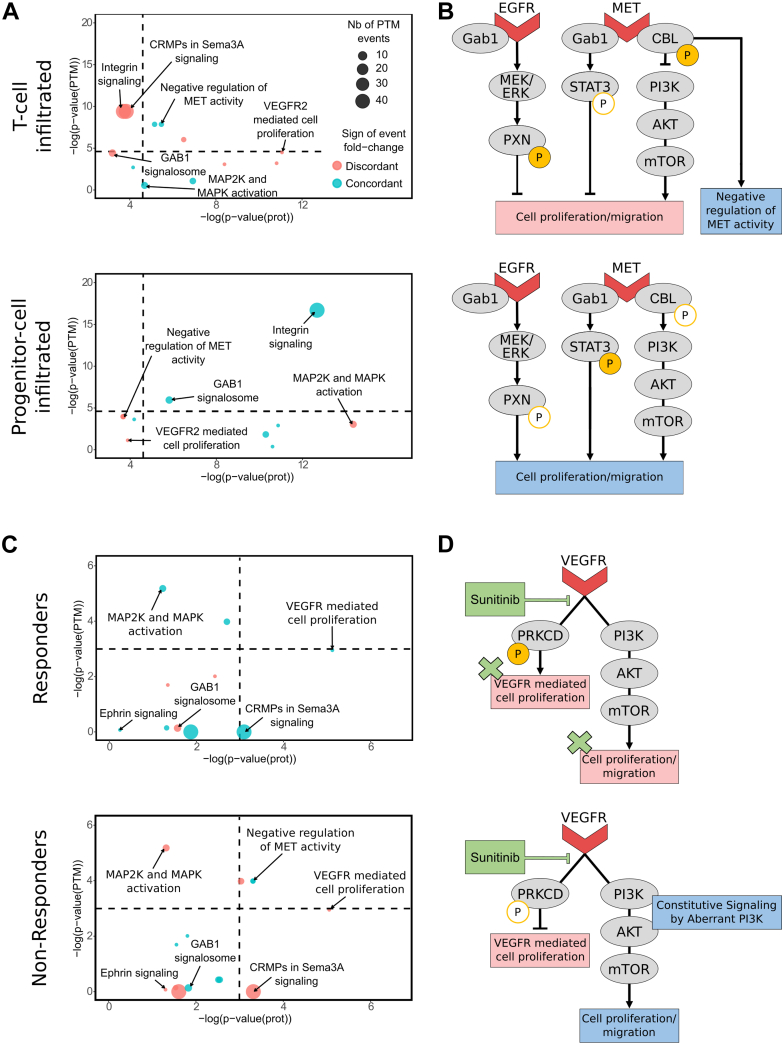
**Biological pathway analysis across sunitinib-treated patient clusters.***A*, pathway significance obtained with expression and phosphorylation data in T-cell–infiltrated and progenitor cell–infiltrated clusters. *B*, selected proteins and pathways involved in progenitor or T-cell infiltration. Annotations concerning delta values and *p* values are presented in Supplemental Fig. S1. *C*, selected proteins and pathways involved in response to sunitinib. In responder patients, cell proliferation pathways are inactivated because of VEGFR inhibition by sunitinib. *D*, pathway significance obtained with expression and phosphorylation data in responder and nonresponder clusters. PTM integration for ligand–receptor interaction inference. PTM, post-translational modification; VEGFR, vascular endothelial growth factor receptor.

We analyzed LRI predictions in the two immune clusters most associated with treatment response: T-cell–infiltrated and progenitor cell–infiltrated tumors, as determined by the authors. We found 3322 unique LRIs involving 381 pathways in the T-cell–infiltrated cluster. In the progenitor cell–infiltrated cluster, we found 3002 unique LRIs and 315 unique pathways. Among them, 167 LRIs and 13 pathways in the T-cell–infiltrated cluster, and 175 and 10 in the progenitor cell–infiltrated cluster, respectively, were involved in phosphorylation events based on position-level information (Supplemental Table S4). Although several pathways were identified as pleiotropic, such as clathrin-mediated endocytosis or integrin signaling pathways, most were cancer related, such as MAP2K and MAPK activation or VEGFR2-mediated cell proliferation pathways (Fig. 6*A*).

Interestingly, the negative regulation of the MET activity pathway was found to be associated with decorin–MET interaction in both clusters based on proteomics data (*p* = 0.04 in progenitor cell–infiltrated cluster, *p* = 0.004 in T-cell–infiltrated cluster). However, two amino acids of a protein expected to be phosphorylated in this pathway, CBL S-483 and S-486, displayed a negative delta value in the progenitor cell–infiltrated cluster (Δ = −29 and −30, *p* = 0.02) and a positive delta value in the T-cell–infiltrated cluster (Δ = 34 and 41, *p* = 0.0003). This suggested an activation of this antitumoral pathway (36) in T-cell–infiltrated samples, which usually show better treatment response than progenitor cell–infiltrated samples (Fig. 6B). These conclusions are concordant with the activation of the protumoral TGF-β pathway found in progenitor cell–infiltrated clusters in the original publication.

Moreover, although the T-cell–infiltrated cluster was expected to exhibit a more antitumoral TME (37, 38), proteomic data predicted activation of the protumoral (39, 40) GAB1 signalosome pathway, which involves dephosphorylation of the PXN protein. However, our phosphoproteomic analysis revealed that PXN, expected to be dephosphorylated at six sites, showed increased phosphorylation levels, with positive delta values ranging from 0.9 to 2.5 (*p* = 0.01). This indicated enhanced PXN phosphorylation within the T-cell–infiltrated cluster, suggesting inactivation of the GAB1 signalosome pathway. Contrary to predictions based on protein expression, these results aligned with the antitumoral profile of the TME.

To investigate whether phosphoproteome information may provide new insights into sunitinib treatment response mechanisms, we compared responder and nonresponder patients. Several cancer-related pathways were predicted in both groups based on expression data. Notably, MAP2K and MAPK activation and VEGFR2-mediated cell proliferation pathways were found in both clusters with high confidence (*p* = 10^−3^ and *p* = 2.10^−4^ for both groups). However, most of the sites expected to be phosphorylated in these pathways (MAPK1-T185, MAP2K2-S226, MAPK3-S202, and PRKCD-T507) harbored negative delta value in the nonresponder group, suggesting that they were less activated in these patients (Fig. 6*C*). On the contrary, these sites displayed positive delta values in the responder cluster, which was coherent with the pathways being active in this group. Sunitinib targets multiple receptor tyrosine kinases, including VEGFR. The pre-existing downregulation of VEGFR pathways in nonresponders suggested that the drug could not effectively modulate the signaling as it did in the responder group. This may account for the resistance to therapy. Moreover, in nonresponder patients, we detected in our first BulkSignalR analysis constitutive signaling mediated by aberrant PI3K that activates cell proliferation and migration pathways independently of VEGFR. Such constitutive signaling could promote tumor growth despite treatment administration (Fig. 6*D*).

## Discussion

Traditional inference and analysis of LRIs largely rely on gene or protein expression. However, this approach overlooks critical dynamic regulatory layers, especially the PTM-dependent activation states of receptors and downstream signaling. Our results show that integrating PTMs in the analysis of LRIs has the potential to improve the accuracy of LRI predictions and to refine the inference of downstream signaling pathway activity. That is, the use of PTM data enhances our understanding of cellular networks.

Our tool can be used to identify and highlight PTMs occurring in LRI-induced pathways. This information is crucial to explore cellular pathway dynamics, and it can also be used to assess pathway activity. As we showed, PTM information can help to discard LRI-associated pathways that would be supported by expression data, although final regulation is operated by specific PTMs (41). Notably, the integration of phosphoproteomics data enabled us to discriminate context-specific activation of pathways in response to the same ligand under different experimental conditions or in different classes of samples. This agrees with previous results demonstrating that signaling specificity is solely dictated not only by ligand identity or receptor expression but also by the post-translational state of the receptor complex (42, 43, 44). For instance, our tool illustrates that phosphorylation at specific regulatory sites of receptor tyrosine kinases can differ strongly between two groups of patients despite upregulated LRI-involved molecules being detected in both cases. The comparison of PTM abundances between clusters allows us to identify which sample group displays PTMs associated with a given cellular pathway and suggests its relative activity state. This is particularly relevant in a clinical or preclinical context, where treatments targeting the same receptor may yield divergent outcomes depending on protein modification status. As presented in our results, and logically, our method seems well adapted to analyze tumor response to a therapy targeting specific PTMs such as kinase inhibitors. One limitation of our method is related to the biased and partial knowledge present in existing databases such as Reactome or PhosphoSitePlus. To overcome this hurdle, the database proposed with this new BulkSignalR extension can be enriched and modified by the user to include new PTM-pathway associations or other types of modifications. The tool also enables using transcriptomics for expression data instead of expression proteomics for LRI prediction, before integrating PTMs, as also proposed by a recent software package named Incytr, which imposes single-cell RNA sequencing for expression data before using bulk phosphoproteomics for the PTM level (14). Our tool can handle both bulk and single-cell expression data in proteomics or transcriptomics.

In conclusion, our results showed that incorporating PTMs into cellular network inference methods gives access to deeper insight into cellular networks.

## Data Availability

Reference lists of PTMs along with their positions on proteins are available in the supplemental data. The extended version of BulkSignalR, including PTM support, is available from GitHub (https://github.com/girouxpierre/PTMSignalR) and the Zenodo repository (https://doi.org/10.5281/zenodo.15882213). The BulkSignalR companion GitHub repository (https://github.com/jcolinge/BulkSignalR) contains an example of application of the previous version. LSCC data were downloaded from the CPTAC Pan-Cancer Data Portal, and ccRCC data were downloaded from ProteomeCentral, both public repositories (see details in the Experimental Procedures section).

## Supplemental data

This article contains supplemental data.

## Conflict of interest

The authors declare no competing interests.
