# Supplementary material for: Post-Transcriptional Modification Integration for Ligand–Receptor Cellular Network Inference
Source: Mol Cell Proteomics. 2025 Dec 19;25(3):101493. doi: 10.1016/j.mcpro.2025.101493 (PMC12933558; doi:10.1016/j.mcpro.2025.101493)

SUPPLEMENTAL MATERIAL

**Post-transcriptional modifications integration for ligand-receptor cellular network inference**

Pierre Giroux^1,2,3^, Morgan Maillard^1,2,3^, Jacques Colinge^1,2,3^

^1^ IRCM, Institut de Recherche en Cancérologie de Montpellier, INSERM U1194, Montpellier, France

^2^ Université de Montpellier, Montpellier, France

^3^ ICM, Institut régional du Cancer de Montpellier, Montpellier, France

Correspondance :

Prof. Jacques Colinge, [jacques.colinge@umontpellier.fr](mailto:jacques.colinge@umontpellier.fr), IRCM, 208 avenue des Apothicaires, F-34298 Montpellier, France

**Supplementary figure 1.** Annotated biological pathway analysis across Sunitinib-treated patient clusters. (**A**) Selected proteins and pathways involved in progenitor (up) or T cell infiltration (bottom). Phosphorylation sites are annotated with their delta values for each identified site. Signaling pathways are annotated with the P-value obtained with the expression-based analysis, as well as with the P-value obtained from the PTM analysis when PTMs are present in the pathway. (**B**) Selected proteins and pathways involved in responder (up) and non-responder clusters (bottom) with their annotation.


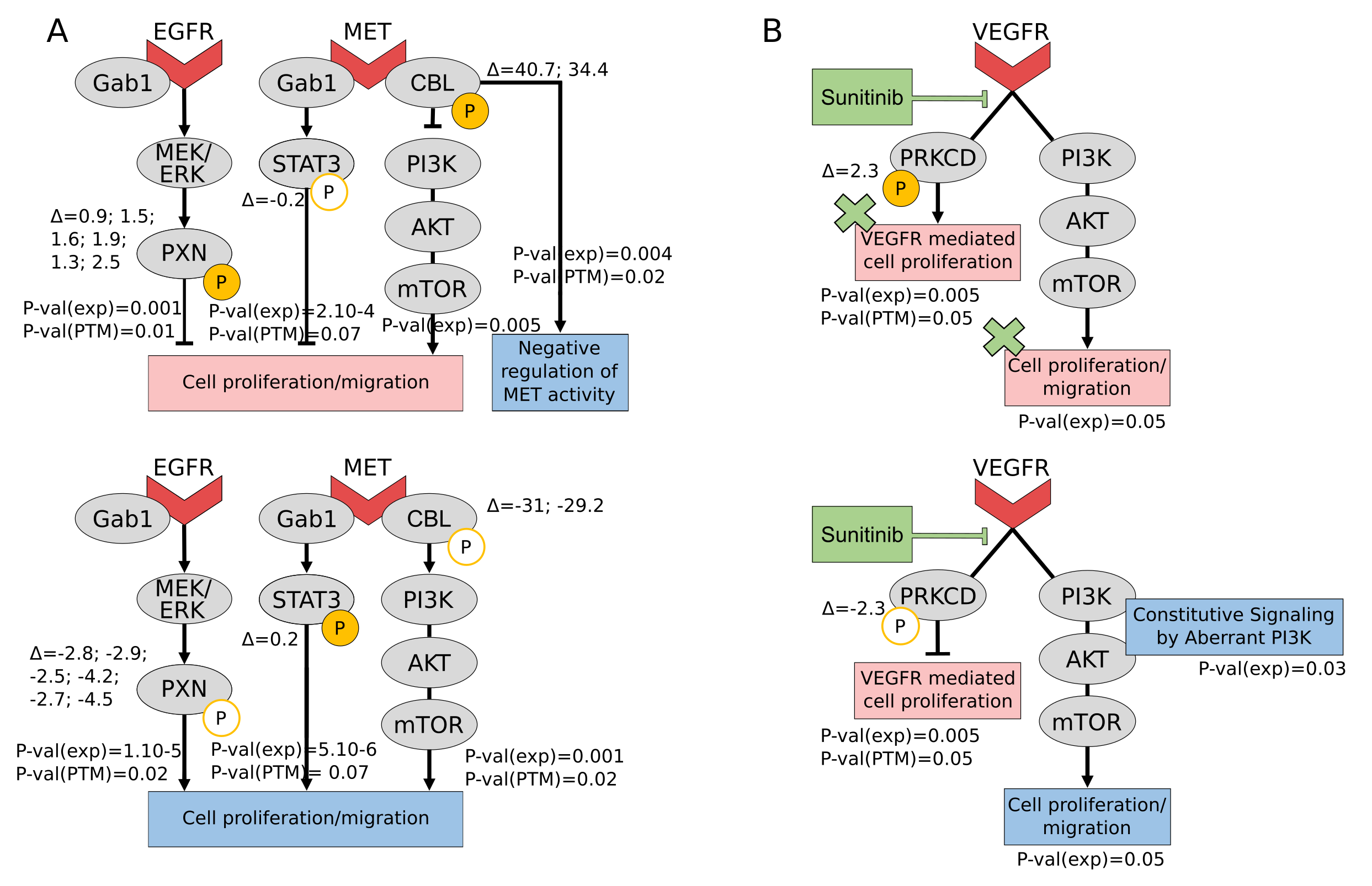

Supplement: Supplementary Figure [file mmc1.docx]
